# The involvement of autotaxin in renal interstitial fibrosis through regulation of fibroblast functions and induction of vascular leakage

**DOI:** 10.1038/s41598-019-43576-x

**Published:** 2019-05-15

**Authors:** Norihiko Sakai, Gretchen Bain, Kengo Furuichi, Yasunori Iwata, Miki Nakamura, Akinori Hara, Shinji Kitajima, Akihiro Sagara, Taito Miyake, Tadashi Toyama, Koichi Sato, Shiori Nakagawa, Miho Shimizu, Shuichi Kaneko, Takashi Wada

**Affiliations:** 1 Division of Nephrology, Kanazawa University Hospital, Kanazawa, 920-8641 Japan; 2 Division of Blood Purification, Kanazawa University Hospital, Kanazawa, 920-8641 Japan; 3 PharmAkea Inc, San Diego, CA 92130 USA; 4 Department of Nephrology and Laboratory Medicine, Institute of Medical, Pharmaceutical and Health Sciences, Kanazawa University, Kanazawa, 920-8641 Japan; 5 Department of System Biology, Institute of Medical, Pharmaceutical and Health Sciences, Kanazawa University, Kanazawa, 920-8641 Japan

**Keywords:** Kidney diseases, Interstitial disease

## Abstract

The accumulation of fibroblasts is a critical step in the development of fibrosis, and lysophosphatidic acid (LPA) promotes fibrosis by regulating multiple fibroblast functions. Autotaxin (ATX) is a key LPA-producing enzyme, and we hypothesized that ATX contributes to the development of renal interstitial fibrosis through LPA-mediated effects on fibroblast functions. In a mouse model of renal interstitial fibrosis induced by unilateral ureteral obstruction (UUO), the levels of renal ATX protein and activity increased with the progression of fibrosis in ligated kidneys, despite concurrent reductions in renal ATX mRNA. UUO enhanced vascular permeability in the renal interstitium, and ATX protein localized to areas of vascular leak, suggesting that vascular leak allowed ATX to enter the renal interstitium. *In vitro* studies showed that ATX induces the migration and proliferation of renal fibroblasts and enhances the vascular permeability of endothelial monolayers. Finally, pharmacological inhibition of ATX partially attenuated renal interstitial fibrosis. These results suggest that during the development of renal fibrosis, ATX accumulates in the renal interstitium and drives fibroblast accumulation and promotes renal interstitial vascular leak, thereby partially contributing to the pathogenesis of renal interstitial fibrosis. Taken together, ATX inhibition may have the potential to be a novel therapeutic strategy to combat renal interstitial fibrosis.

## Introduction

Fibrosis is a final common pathway for many chronic diseases, resulting in end-stage organ failure. Despite varied etiologies, renal diseases develop the shared pathologic features of glomerulosclerosis and renal interstitial fibrosis as they progress to end-stage renal failure^1,2^. Of these characteristic pathological features, the extent of renal interstitial fibrosis has been found to determine the prognosis of renal diseases independent of their etiologies^3^. Histologically, renal interstitial fibrosis is characterized by fibroblast accumulation and increased extracellular matrix deposition causing the disruption of normal renal architecture and homeostasis^4,5^. The pathogenic mechanisms driving fibroblast accumulation and activation in renal interstitial fibrosis remain to be determined.

Lysophosphatidic acid (LPA) is an important bioactive lipid that exerts diverse biological effects on many cell types and tissues^6,7^. LPA mediates various cellular responses through interactions with specific G protein-coupled receptors (GPCRs). To date, at least six cell-surface GPCRs that respond specifically to LPA have been identified in mammalian cells^8^. Of these, we and others have demonstrated that LPA signaling through LPA_1_ significantly contributes to the pathogenesis of fibrosis in various organs by promoting the accumulation of activated fibroblasts^9–15^. Increased LPA concentrations have been reported in biological fluids sampled from organs developing fibrosis, such as bronchoalveolar lavage fluid recovered from the lungs of patients with idiopathic pulmonary fibrosis, or from mice in the bleomycin model of pulmonary fibrosis^9^. These findings suggest that LPA may play a pivotal role in the development of organ fibrosis, and that the inhibition of LPA production may be a novel therapeutic approach for fibrotic diseases.

Autotaxin (ATX; ENPP2) is one of seven mammalian ectonucleotide pyrophosphatases/phosphodiesterases, and was originally isolated from the supernatants of melanoma cells as an autocrine motility factor^16,17^. ATX functions as a lysophospholipase D, generating LPA by the cleavage of the choline head group from lysophosphatidylcholine (LPC), an abundant plasma phospholipid. The ATX pathway appears to be responsible for the majority of extracellular LPA present in the circulation, since plasma LPA levels in mice heterozygous for an ATX-null allele are one half of those present in wild-type mice^18,19^. However, the enzymatic pathway(s) responsible for LPA production in many organs and tissues remains to determined. An alternative and ATX-independent pathway of LPA production has been identified in which LPA is produced from phosphatidic acid by phospholipase A_1_ or phospholipase A_2_ family members, including Lipase H (LIPH)^20^. Recent studies focusing on the role of ATX in organ fibrosis have revealed that ATX is required for the development of dermal and hepatic fibrosis in mice^21–23^. In contrast, pulmonary fibrosis induced by bleomycin in mice was not protected by the inhibition of ATX activity^24^. Whether ATX contributes to the pathogenesis of renal interstitial fibrosis has yet to be investigated.

To more fully understand the role of ATX in renal interstitial fibrosis, we examined the contribution of ATX to the pathogenesis of fibroblast accumulation and renal interstitial fibrosis. Here we found that the levels of renal ATX protein and activity increases during the development of renal interstitial fibrosis in the unilateral ureteral obstruction (UUO) model due to increased renal vascular permeability, rather than from local production. Mechanistically, we demonstrated that ATX induces the migration, proliferation and differentiation of renal fibroblasts, all of which could contribute to fibroblast accumulation in fibrotic kidneys, and that ATX induces increased permeability of endothelial cell monolayers. In addition, pharmacological inhibition of ATX partially attenuated renal interstitial fibrosis as well as fibroblast accumulation induced by UUO. Taken together, our findings demonstrate that ATX might have the potential to be a therapeutic target for renal interstitial fibrosis.

## Results

### UUO induces renal interstitial fibrosis accompanied with renal fibroblast accumulation and proliferation

**Figure 1:**
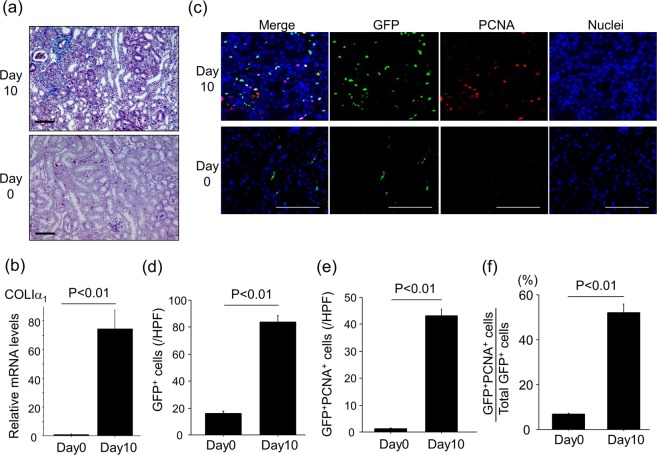
UUO-induced renal interstitial fibrosis is accompanied by renal fibroblast accumulation and proliferation. (**a**) Representative Mallory Azan-stained kidney sections following UUO (magnification x 200). Bars, 100 μm. (**b**) Relative mRNA levels of *Col1a1* in kidneys (n = 5 mice/group). ΔΔCT method was used to calculate relative gene expression of *Col1a1* with GAPDH being the internal control. Data are expressed as mean ± SEM. (**c**) Accumulation of proliferating fibroblasts (GFP^+^PCNA^+^) ten days after UUO. GFP-stained renal sections were obtained from COL-GFP mice. Representative tissue sections stained with anti-GFP antibody/anti-PCNA antibody are shown. Bars, 100 μm. (**d**) Numbers of GFP^+^ cells in the kidney are expressed as the mean number ± SEM per HPF (n = 5 mice/group). (**e**) Numbers of renal GFP^+^PCNA^+^ cells (proliferating fibroblasts) are expressed as mean number ± SEM per HPF. (**f**) Percentages of renal fibroblasts that are proliferating (GFP^+^PCNA^+^ cells/total GFP^+^ cells).

Progressive renal interstitial fibrosis was induced by ureteral ligation, as demonstrated by increases in Mallory-Azan staining of renal collagen (Fig. 1a) and renal mRNA levels of COLIα_1_ (Fig. 1b). Accumulation of collagen-producing fibroblasts is recognized as a critical step for progressive fibrosis^25,26^. Therefore, we examined the expansion of the fibroblast pool during the development of renal interstitial fibrosis using COLI-GFP mice subjected to the UUO model. In these COLI-GFP mice, all fibroblasts can be identified by their transgenic expression of enhanced GFP driven by the promoter of the α_2_ chain of type I pro-collagen^27^. We consequently identified fibroblasts in these experiments by immunostaining renal sections for GFP-expressing cells. As demonstrated in the representative sections shown in Fig. 1c, UUO induced a marked renal accumulation of GFP^+^ fibroblasts (Fig. 1d). To specifically identify proliferating fibroblasts, we double-stained renal sections with anti-PCNA antibody and anti-GFP antibody. As demonstrated in the representative sections in Fig. 1c, the number of proliferating fibroblasts (GFP^+^PCNA^+^ cells) was increased by UUO (Fig. 1e). The percentage of proliferating fibroblasts among total fibroblasts (the percentage of GFP^+^PCNA^+^ cells among total GFP^+^ cells) was also significantly increased in ligated kidneys (Fig. 1f). These data suggest that UUO induced the expansion of the collagen-producing fibroblast pool through the induction of fibroblast proliferation during the course of renal interstitial fibrosis.

### Renal ATX protein and activity are increased with the progression of renal interstitial fibrosis

**Figure 2:**
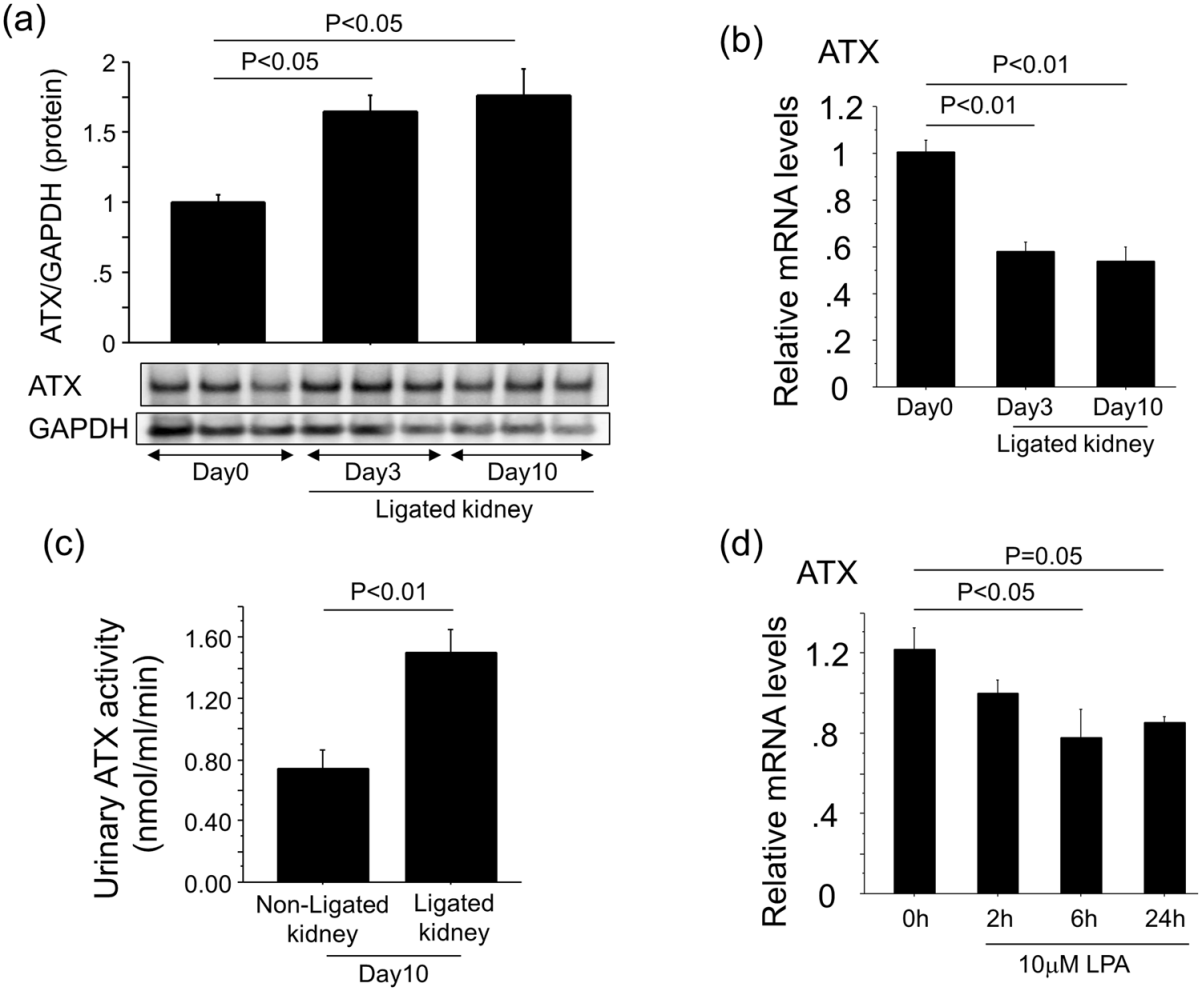
Renal ATX protein levels increase with the progression of renal interstitial fibrosis. (**a**) The expression of ATX protein in whole kidney lysates at day 0, 3 and 10 post-UUO. Quantification was performed with Image J software and data are expressed as mean dots of ATX bands relative to dots of GAPDH bands ± SEM (n = 3 mice/group). (**b**) Relative mRNA levels of ATX in whole kidney lysates obtained from mice following UUO. ΔΔCT method was used to calculate relative gene expression of ATX with GAPDH being the internal control. (n = 5 mice/group). (**c**) ATX activity in urine obtained from ligated kidneys and non-ligated kidneys (bladder). Data are expressed as mean ± SEM concentrations of liberated choline per minute. (n = 4–5 mice/group). (**d**) Relative mRNA levels of ATX in renal fibroblasts in response to LPA. ΔΔCT method was used to calculate relative gene expression of ATX with β2MG being the internal control. Data are expressed as mean ± SEM. (n = 2 cell preparations/group). Data are expressed as mean ± SEM.

**Figure 3:**
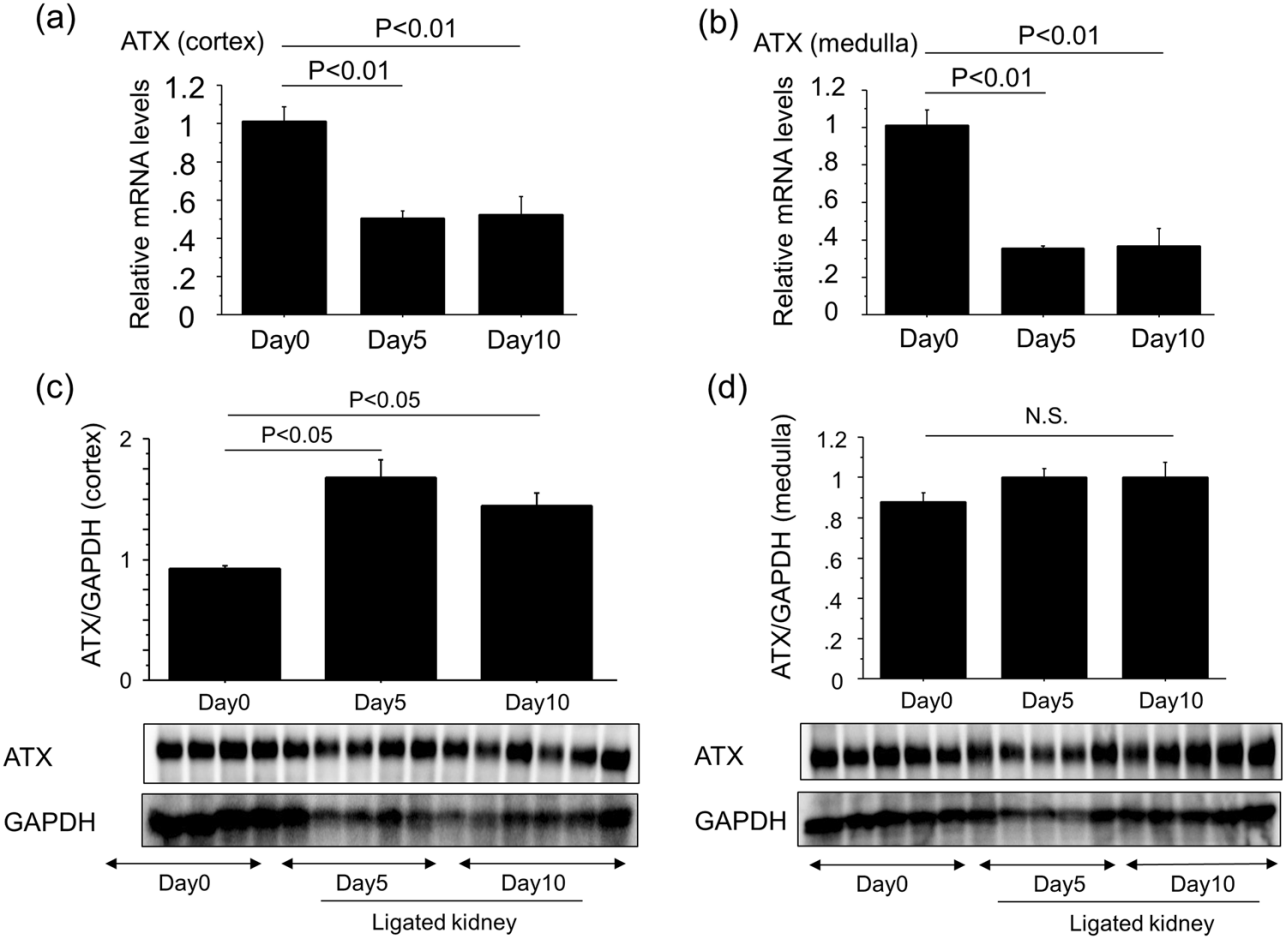
ATX protein levels in the cortex of ligated kidneys increase with the progression of renal interstitial fibrosis. (**a**,**b**) Relative mRNA levels of ATX in kidneys obtained from cortex (**a**) and medulla (**b**) at day 0, 5 and 10 post-UUO (n = 5 mice/group). ΔΔCT method was used to calculate relative gene expression of ATX with GAPDH being the internal control. Data are expressed as mean ± SEM. (**c**,**d**) The expression of ATX protein in kidney lysates obtained from cortex (**c**) and medulla (**d**) at day 0, 5 and 10 post-UUO. Quantification was performed with Image J software and data are expressed as mean dots of ATX bands relative to dots of GAPDH bands ± SEM (n = 5 mice/group).

Renal LPA concentrations have been reported to be increased in the UUO model of renal interstitial fibrosis model^13,28^. We therefore examined if renal ATX production was also up-regulated as a LPA-producing pathway in this model. Accompanied with the progression of renal interstitial fibrosis, the protein levels of ATX increased in ligated whole kidneys (Fig. 2a), whereas ATX mRNA levels in ligated whole kidneys decreased with the progression of renal interstitial fibrosis (Fig. 2b). In addition, ATX activity in urine obtained from the pelvis of ligated kidneys at day 10 was higher than that in urine taken from the bladder, which came from non-ligated kidneys (Fig. 2c). The stimulation of primary mouse renal fibroblasts by LPA suppressed ATX mRNA expression (Fig. 2d). Similarly, ATX mRNA expression in both the cortex and medulla of ligated kidneys decreased after UUO (Fig. 3a,b), whereas ATX protein increased especially in the cortex of ligated kidneys (Fig. 3c,d). These results suggest that the higher amount of ATX protein in ligated kidneys may not be related to the local transcriptional induction of ATX in the ligated kidneys.

### Interstitial ATX is increased in areas of UUO-induced vascular leak

**Figure 4:**
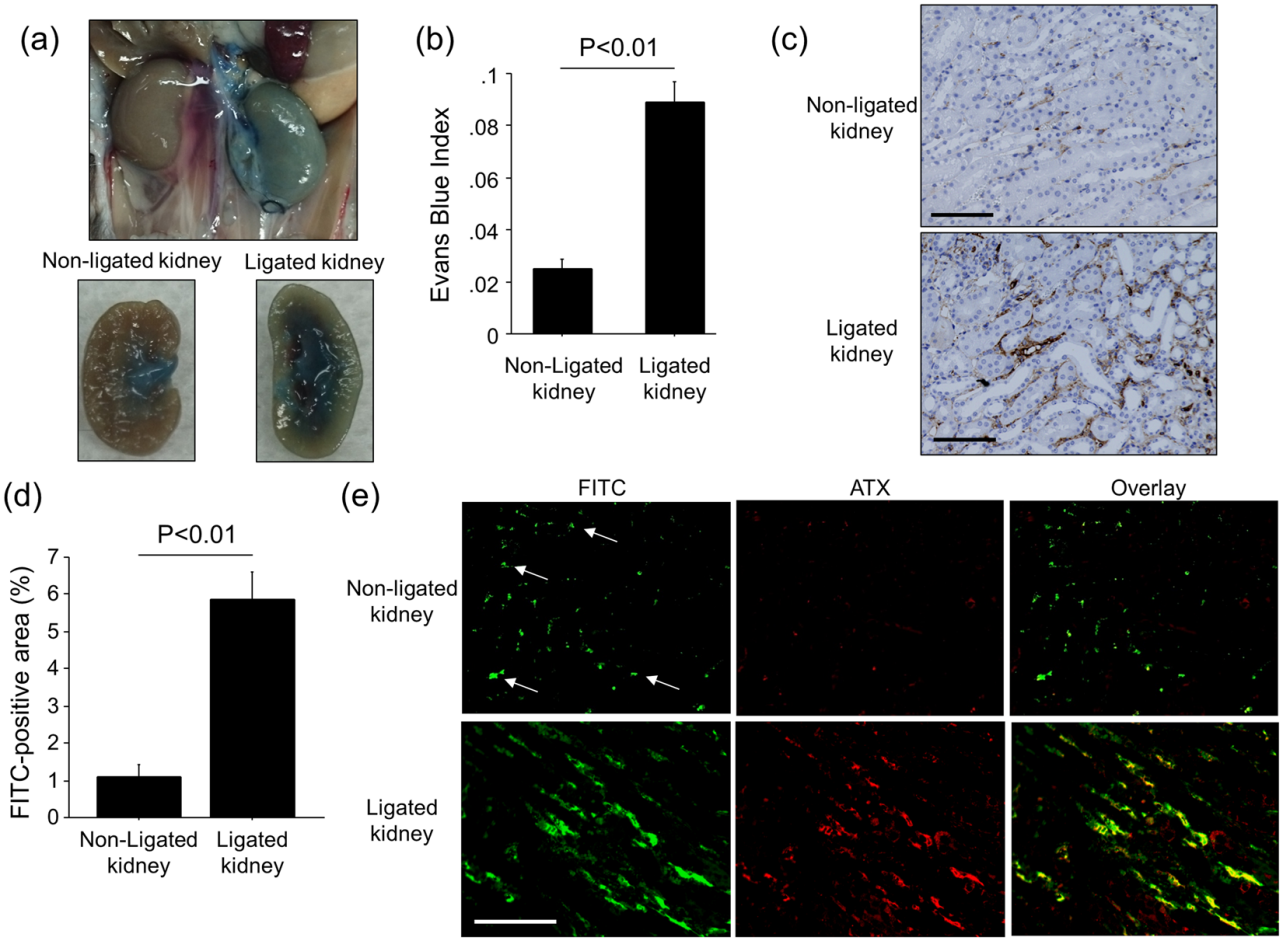
UUO-induced vascular leak co-localizes with interstitial ATX. (**a**) Estimation of renal vascular permeability by extravasation of Evans Blue dye (EBD). Gross appearance of representative kidneys 10 days after UUO surgery and 30 min after Evans Blue injection. (**b**) Indices of Evans Blue leakage into non-ligated kidneys and ligated kidneys (n = 5 mice/group). Data are expressed as mean Evans Blue index ± SEM. (**c**,**d**) The leaked area of injected FITC-Dextran (500 kDa) in kidneys obtained 10 days after UUO (n = 5 mice/group). Data are expressed as mean ± SEM. (**e**) Co-localization of injected FITC-Dextran (green) and ATX (red) in kidneys. Representative tissue sections are shown (magnification x 200). Arrows indicate the intact blood vessels. Bars, 100 μm.

ATX is present in the circulation, yet this protein is not filtered in the glomerulus since its molecular weight is high (~125 kDa)^29^. Therefore, we hypothesized that increased renal vascular permeability in the interstitium induced by UUO enabled circulating ATX to enter the interstitium of ligated kidneys. To quantify renal vascular permeability, albumin-binding EBD was injected into mice via tail vein eight days after ureteral ligation. The injection of EBD was followed by the injection of saline to wash out any dye remaining in the vasculature. As shown in Fig. 4a, the ligated kidney retained much more EBD after saline vascular washout as compared to the non-ligated kidney, indicative of albumin-bound dye leaking into the interstitium of the ligated kidney. Quantification of EBD levels in renal tissues confirmed that ligated kidneys retained significantly more dye than non-ligated kidneys (Fig. 4b), suggesting that ureteral ligation increased vascular permeability in ligated kidneys during the development of renal interstitial fibrosis. To further confirm an increase in vascular permeability, FITC-conjugated dextran (500 kDa) was injected via tail vein eight days after ureteral ligation to assess where the vascular leak occurred. Immunohistochemical studies demonstrated marked FITC-dextran deposition in the renal interstitial space of fibrotic kidneys, suggesting that UUO induced vascular leak in the interstitium of fibrotic kidney (Fig. 4c,d). We also determined ATX localization by immunofluorescent staining following FITC-dextran injection. As shown in Fig. 4e, ATX co-localized with FITC-dextran in the interstitial space of ligated kidneys. These results suggest that the increase in ATX protein we observed in fibrotic kidneys was due, at least in part, to increased renal vascular permeability that resulted in ATX entering into the renal interstitium.

### ATX-LPA_1_ signaling regulates renal fibroblasts migration, proliferation and myofibroblast differentiation

**Figure 5:**
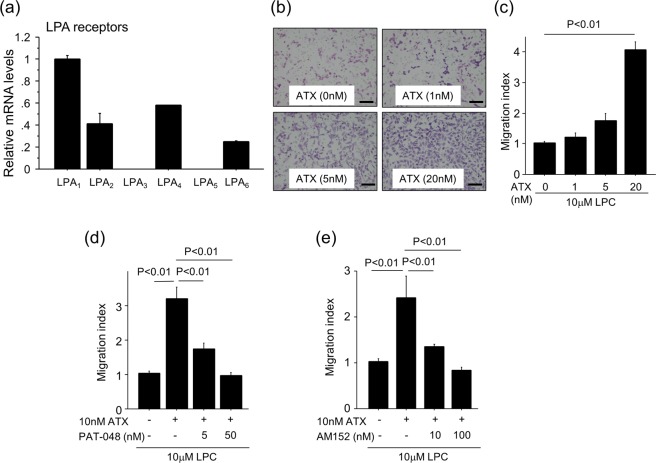
ATX-LPA_1_ signaling regulates renal fibroblast migration. (**a**) Relative mRNA levels of LPA receptors in renal fibroblasts. ΔΔCT method was used to calculate relative gene expression of LPA receptors with β2MG being the internal control. Data are expressed as mean ± SEM. (**b**) Representative pictures of migrated renal fibroblasts in response to 10 μM LPC with indicated concentrations of recombinant ATX. Bars, 100 μm. (**c**) The addition of ATX to LPC enhanced renal fibroblast migration in an ATX concentration-dependent manner. (n = 3 cell preparations/group). (**d**,**e**) Pre-treatment with PAT-048 (**d**) or AM152 (**e**) suppressed renal fibroblast migration induced by ATX and LPC in a concentration-dependent manner. (n = 3 cell preparations/group). Data are expressed as mean ± SEM.

**Figure 6:**
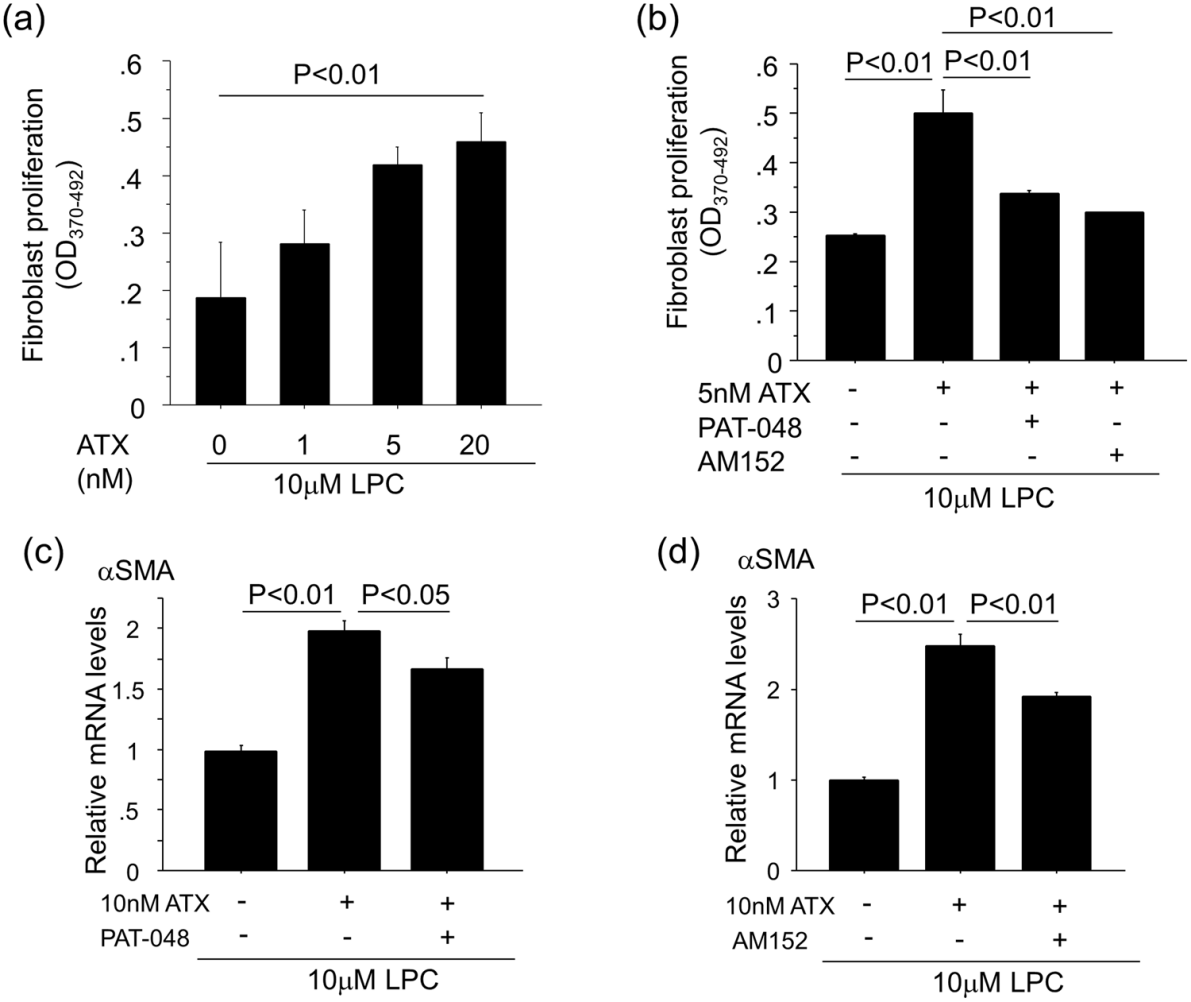
ATX-LPA_1_ signaling regulates renal fibroblasts migration, proliferation and myofibroblast differentiation. (**a**) The addition of ATX and LPC stimulated the proliferation of renal fibroblasts in an ATX dose-dependent manner. (n = 3 cell preparations/group). (**b**) Renal fibroblast proliferation induced by the addition of ATX with LPC was inhibited by 5 nM PAT-048 and 10 nM AM152, respectively. Data are expressed as mean ± SEM. (**c**,**d**) αSMA expression in renal fibroblasts induced by the addition of ATX with LPC was inhibited by 50 nM PAT-048 (**c**) and 100 nM AM152 (d) (n = 3 cell preparations/group). ΔΔCT method was used to calculate relative gene expression of αSMA with β2MG being the internal control. Data are expressed as mean ± SEM.

We performed a series of *in vitro* experiments using primary mouse renal fibroblasts to investigate the mechanisms by which ATX regulates the accumulation of fibroblasts. We observed the expression of all LPA receptors in renal fibroblasts with LPA_1_ having the highest level of expression (Fig. 5a), as was previously observed in lung fibroblasts^9^. Assays of renal fibroblast migration showed that when these cells were exposed to recombinant ATX along with its substrate, LPC, the migration of these fibroblasts was enhanced in an ATX concentration-dependent manner (Fig. 5b,c). Pre-treatment with the selective ATX inhibitor, PAT-048, suppressed renal fibroblast migration in response to ATX, with approximately 60% inhibition at 5 nM PAT-048 and full inhibition of migration at 50 nM PAT-048 (Fig. 5d). The LPA_1_-selective small molecule antagonist, AM152, also inhibited renal fibroblast migration in a concentration-dependent manner with 100 nM demonstrating full inhibition (Fig. 5e). Taken together, these data suggest that ATX induces renal fibroblast migration by producing LPA, which then signals through the LPA_1_ expressed by these cells. We then examined the effects of ATX activity on renal fibroblast proliferation. The incubation of renal fibroblasts with ATX along with LPC for 24 hours stimulated the proliferation of these cells in an ATX concentration-dependent manner (Fig. 6a). The renal fibroblast proliferation induced by ATX was significantly inhibited by pre-treatment with 5 nM PAT-048 and 10 nM AM152 (Fig. 6b), suggesting that ATX-LPA_1_ signaling is an important pathway for renal fibroblast proliferation as well. Furthermore, the stimulation of renal fibroblasts with ATX along with LPC induced alpha smooth muscle actin (αSMA) expression, which is a marker of myofibroblasts (Fig. 6c,d). In addition, αSMA expression induced by ATX along with LPC was significantly reduced by treatment with 50 nM PAT-048 and 100 nM AM152 (Fig. 6c,d). These data demonstrate that ATX can induce migration, proliferation and differentiation to myofibroblasts of renal fibroblasts through LPA_1_ signaling.

### ATX regulates vascular permeability of endothelial monolayers

**Figure 7:**
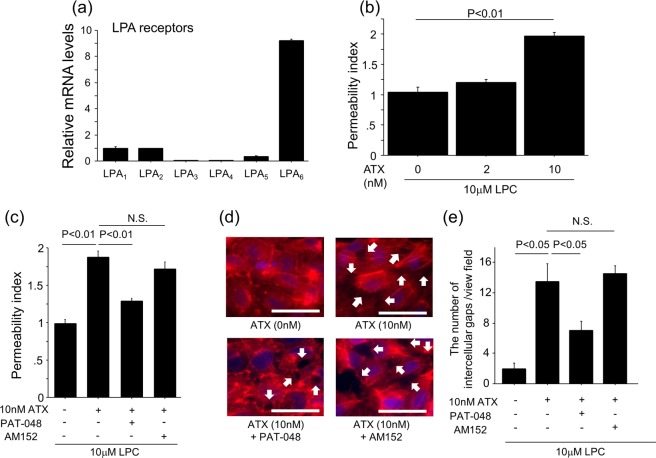
ATX regulates HUVEC permeability via a LPA_1_-independent mechanism. (**a**) Relative mRNA levels of LPA receptors in HUVECs. ΔΔCT method was used to calculate relative gene expression of LPA receptors with β2MG being the internal control. Data are expressed as mean ± SEM. (**b**) The addition of ATX and LPC to HUVECs enhances permeability in an ATX concentration-dependent manner. (n = 3 cell preparations/group). (**c**) Pre-treatment with 5 nM PAT-048, but not 10 nM AM152, suppresses endothelial cell permeability induced by ATX and LPC (n = 3 cell preparations/group). Data from (**b**,**c**) are expressed as mean ± SEM. (**d**) Actin polymerization was visualized by immunocytochemical staining for phalloidin in HUVECs that had been incubated in media containing 0.5% FBS for 2 hours and then stimulated for 30 min with 10 nM ATX added to 10 μM LPC or 10 μM LPC alone. HUVECs were pre-treated with 5 nM PAT-048 or 10 nM AM152 for 30 min. Arrows indicate intercellular gaps. Bars, 100 μm. All images were captured using identical exposure settings. (**e**) The number of intercellular gaps induced by the addition of ATX with LPC was inhibited by 5 nM PAT-048, but not 10 nM AM152. Four different fields were counted. Data are expressed as mean ± SEM.

LPA has been reported to induce cell contraction via regulation of actin cytoskeleton^30^. We therefore hypothesized that the ATX-LPA axis contributes to the vascular leak through LPA-induced actin cytoskeleton rearrangement of vascular endothelial cells. To test this hypothesis, we performed *in vitro* vascular permeability assays using HUVECs. HUVECs express several LPA receptors, with LPA_6_ showing the highest levels of expression (Fig. 7a). The addition of ATX and LPC to HUVECs enhanced the vascular permeability of the endothelial cell monolayers in an ATX concentration-dependent manner (Fig. 7b). Pre-treatment of the HUVECs with PAT-048 suppressed vascular permeability induced by ATX, whereas pre-treatment with the LPA_1_-selective antagonist AM152 had no significant effect (Fig. 7c). We also examined the effects of ATX on endothelial monolayers by visualizing the actin cytoskeleton in HUVECs. In the resting state, HUVECs actin cytoskeletons formed cortical rings which facilitate the formation of tight intercellular adhesions (Fig. 7d). Stimulation of HUVECs with ATX and LPC induced the reorganization of the cytoskeletons into transcellular actin stress fibers which leads to cell contraction and the formation of intercellular gaps (Fig. 7d). The number of ATX-induced intercellular gaps was attenuated by pre-treatment with PAT-048 but not AM152 (Fig. 7e). Taken together, these data indicate that ATX also regulates the development of vascular leak through endothelial actin cytoskeleton rearrangement and this occurs in an LPA_1_-independent manner, in contrast to the ATX-mediated regulation of fibroblast migration and proliferation.

### Pharmacological inhibition of ATX partially attenuates renal interstitial fibrosis and fibroblast accumulation

**Figure 8:**
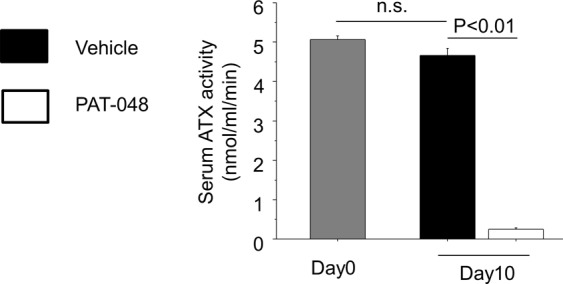
*In vivo* effect of PAT-048 on serum ATX activity. ATX activity in serum obtained from mice before ureteral ligation (Day0) and ten days after ureteral ligation (Day10) treated with PAT-048 or vehicle (n = 5 mice/group). Data are expressed as mean ± SEM concentrations of liberated choline per minute. n.s.; not significant.

**Figure 9:**
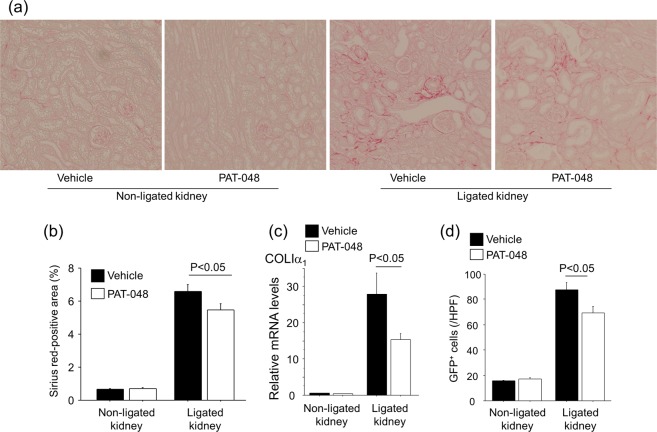
Pharmacological inhibition of ATX partially protects mice from UUO-induced renal interstitial fibrosis. (**a**) Representative Sirius red-stained kidney sections of vehicle-treated and PAT-048-treated mice (magnification × 200). Bars, 100 μm. (**b**) Quantitative analysis of Sirius red staining in kidney sections (Day 10, n = 5 mice/group). (**c**) Relative mRNA levels of *Col1a1* in renal cortex obtained from vehicle-treated and PAT-048-treated mice following UUO (Day 10, n = 5 mice/group). ΔΔCT method was used to calculate relative gene expression of *Col1a1* with GAPDH being the internal control. Data are expressed as mean ± SEM. (**d**) Numbers of GFP^+^ cells in the kidney from mice treated vehicle or PAT-048 (Day 10, n = 5 mice/group). Data are expressed as the mean number ± SEM per HPF.

To investigate the role of ATX in the development of renal interstitial fibrosis, we administered PAT-048 or vehicle to COLI-GFP mice subjected to the UUO. At first, we checked the *in vivo* pharmacodynamics activity of PAT-048. Serum in mice treated with PAT-048 showed 94.5% reduction of ATX activity compared to that in treated with vehicle, suggesting that the dose of PAT-048 administered in this study was sufficient to inhibit ATX activity in this *in vivo* model (Fig. 8). In addition, as shown in Fig. 9a,b, mice treated with PAT-048 were partially protected from renal interstitial fibrosis as indicated by a small but significant reduction in Sirius red staining. The expression of COLIα_1_ in renal cortex was also partially reduced by the administration of PAT-048 (Fig. 9c). Moreover, UUO-induced accumulation of GFP^+^ fibroblasts were partially reduced by treatment with PAT-048 (Fig. 9d). These data suggest that ATX contributes to the expansion of the collagen-producing fibroblast pool during the course of renal interstitial fibrosis.

## Discussion

**Figure 10:**
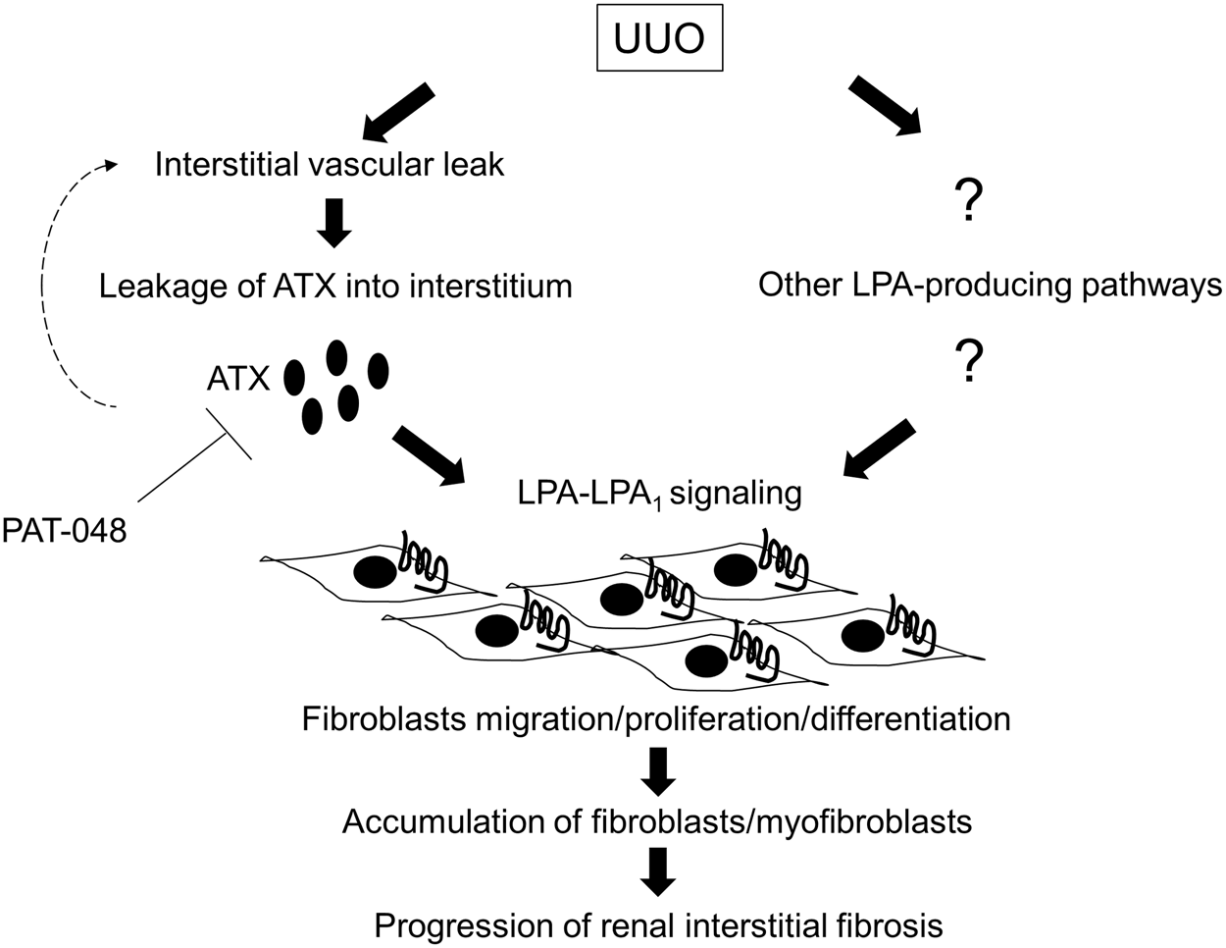
Proposed schema for the mechanism of renal interstitial fibrosis regulated by ATX-LPA-LPA_1_ signaling dependent on interstitial vascular leak. Interstitial vascular leak induced by UUO causes the leakage of ATX into the interstitium. The interstitial ATX then catalyzes the production of LPA which further enhances interstitial vascular leak, thus creating a vicious cycle of LPA-driven renal vascular leak. Additionally, the produced LPA contributes to the accumulation of fibroblasts/myofibroblasts by driving fibroblast migration, proliferation and differentiation dependent on LPA_1_. Other pathways would also be involved in LPA production. Besides ATX-LPA_1_ signaling, multiple pathways producing LPA could be targets to treat renal interstitial fibrosis.

In this study, we found that ATX was partially required for the development of renal interstitial fibrosis. Renal ATX protein and activity levels increase with progression of renal interstitial fibrosis, and pharmacological antagonism of ATX reduces the accumulation of fibroblasts in ligated kidneys and partially protects mice from renal interstitial fibrosis in the UUO model. ATX likely promotes the accumulation of fibroblasts by inducing both their migration, proliferation and differentiation, as shown by the *in vitro* induction of these activities in primary renal fibroblasts exposed to ATX and LPC and the suppression of migration, proliferation and differentiation of renal fibroblasts by pharmacological inhibition of ATX. Based on these data, we conclude that ATX contributes to the development of renal interstitial fibrosis, at least in part, by promoting the accumulation of fibroblasts (Fig. 10).

We also found evidence that vascular leak induced during the development of UUO-mediated renal interstitial fibrosis was responsible for the increase in renal ATX protein and activity rather than local synthesis of new ATX mRNA in the kidney. The induction of vascular leak has been recognized as a critical step in the pathogenesis of pulmonary fibrosis as demonstrated by the exacerbation of vascular leak augmented fibrosis in the bleomycin model of pulmonary fibrosis, potentially due to pro-fibrotic signaling by coagulation proteases such as thrombin that are activated by vascular leak-induced extravascular coagulation^9,31,32^. As observed in this study, increased renal interstitial vascular permeability has been previously reported in UUO-induced renal fibrosis^33,34^. We also found evidence that, besides its accumulation being regulated by vascular leakage, ATX itself contributes to increased vascular permeability. We show here that exposure of HUVEC monolayers to ATX and LPC induces actin cytoskeleton rearrangement in these endothelial cells and results in the formation of intercellular gaps. Further, pharmacological inhibition of ATX blocked these effects on HUVECs. Taken together, our findings suggest that increases in renal ATX levels and vascular permeability form a vicious cycle in the development of renal fibrosis, in which vascular leak allows ATX to enter the renal interstitium, and this increase in ATX levels in turn further increases renal interstitial vascular permeability. Based on these data, we conclude that ATX contributes to the development of renal interstitial fibrosis by promoting renal interstitial vascular leak as well as by promoting the accumulation and differentiation of fibroblasts (Fig. 10). However, the effect of ATX inhibition on renal interstitial fibrosis was only partial in this study. Recently, several ATX-independent pathways, such as phospholipase A_1_ or phospholipase A_2_ family members, have also been reported to be able to produce LPA. In this study, PAT-048 administration showed 94.5% reduction of ATX activity in serum, however, PAT-048 only partially suppressed renal interstitial fibrosis, suggesting that other pathways would also be important for LPA production in this model. Therefore, multiple pathways producing LPA could be targets to treat renal interstitial fibrosis (Fig. 10). In addition, the question regarding the cells responsible for ATX and LPA production remains to be investigated. Taken together, further studies will be required to clarify the spectrum of LPA-producing pathways that contribute to the development of renal fibrosis.

We and others have noted that LPA signaling through its receptors is required for the development of fibrosis in multiple organs^9–15^, thus making inhibition of LPA production and/or inhibition of its receptors a promising new strategy for anti-fibrotic therapies. Among the LPA receptors, LPA_1_ is highly expressed on fibroblasts, and its signaling in these cells can mediate the over-exuberant fibroblast wound-healing response to tissue injury that contributes to fibrosis^9–15,35^. In this study, we showed that ATX regulates pro-fibrotic functions of renal fibroblasts, including migration and proliferation, and promotes the accumulation of fibroblasts through LPA-LPA_1_ signaling. LPA receptors other than LPA_1_ have also been observed to contribute to the pathogenesis of organ fibrosis. LPA_2_ has been reported to contribute to the development of renal and pulmonary fibrosis through the activation of transforming growth factor (TGF)-β signaling in epithelial cells^25,36^. LPA_6_ has been recently reported to be highly expressed on HUVECs and to mediate LPA-induced vascular permeability^37^. Consistent with this observation, we observed that when HUVECs were exposed to ATX and LPC, inhibiting LPA production with a small molecule ATX inhibitor was able to suppress the resulting increase in permeability, whereas treatment with an LPA_1_ antagonist was not. These results differ from prior observations in pulmonary fibrosis, in which vascular leak induced in the lungs during the development of fibrosis was markedly attenuated in LPA_1_-deficient mice^9^, suggesting that LPA-induced vascular permeability may be mediated by different LPA receptors in different organ vascular beds. One limitation of this study was the use of HUVECs, as opposed to renal interstitial endothelial cells, to evaluate the *in vitro* effects of ATX on vascular leakage. Future studies using techniques to isolate renal interstitial endothelial cells will be required to evaluate the effects of ATX on this relevant cell type. In addition, ATX/LPA has been reported to be essential for blood vessel formation during embryonic development and angiogenesis during tumorigenesis and tumor metastasis. Therefore, it can’t be excluded that ATX recruits to the obstructed kidney and rebuilds or repairs the local blood vessels. Further studies using endothelial cell-specific LPA receptor deficient mice could also be performed to clarify these mechanisms in future.

Recent studies have revealed mechanisms by which ATX can catalyze LPA production in local microenvironments. ATX has several domains: two consecutive somatomedin B-like domains (SMB1 and SMB2), a catalytic domain, and a nuclease-like domain^38^. Of these, the SMB domains of ATX are homologous to the SMB domains of vitronectin, which are known to mediate protein-protein interactions^39^. It has been proposed that the SMB domains of ATX bind cell surface integrins, enabling integrin-expressing cells to bind ATX, produce localized LPA, and promote directional cell migration^40^. We found UUO-induced vascular leakage localized ATX to the renal interstitium, the site at which renal fibrosis develops and expands. We hypothesize that when circulating ATX enters the renal interstitium due to the increased vascular permeability, it binds to integrins expressed on the resident cells and stimulates local production of LPA. This localized production of LPA in turn drives the migration and proliferation of renal fibroblasts that contributes to the development of renal interstitial fibrosis. Of note, LPA itself has been reported to function as a suppressor of ATX expression through negative feedback regulation^41^. We also found that the stimulation of primary mouse renal fibroblasts by LPA suppressed ATX mRNA expression. Such negative feedback regulation induced by high local renal production of LPA could account for the reductions in ATX mRNA expression we observed in ligated kidneys. Further investigations will be required to clarify the precise mechanisms of ATX mRNA regulation.

In summary, we have shown a causal link between ATX and renal interstitial fibrosis, by demonstrating that ATX is partially required for the development of this pathology in the UUO model. In this study, we have contributed new findings and ideas that following ureteral ligation, (1) ATX enters the renal interstitium due to increased permeability of the vasculature, and then (2) localized ATX in renal interstitial space drives fibroblast accumulation in the interstitium by stimulating fibroblast migration and proliferation through ATX-LPA-LPA_1_ receptor signaling. Furthermore, (3) interstitial ATX further exacerbates renal interstitial vascular leak through LPA-LPA receptors expressed on endothelial cells other than LPA_1_, causing more ATX to enter the renal interstitium. Accordingly, a vicious cycle regulated by ATX is formed to amplify fibrosis. The strategy to suppress LPA production could be effective due to its ability to inhibit both the pro-fibrotic effects of LPA on fibroblasts mediated by LPA_1_ and the pro-fibrotic effects of LPA on endothelial cells mediated by other LPA receptor(s). Besides ATX signaling, multiple pathways producing LPA could be targets to treat renal interstitial fibrosis.

## Materials and Methods

### Reagents and cells

1-oleoyl-LPC (18:1) was purchased from Avanti Polar Lipids (Alabaster, AL, USA) and diluted in Dulbecco’s Modified Eagle’s Medium (Lonza, Walkersville, MD, USA) including 0.1% bovine serum albumin (Sigma Aldrich, St. Louis, MO, USA). The selective ATX inhibitor PAT-048^21,22,24^ and the LPA_1_-selective small molecule antagonist AM152 (Example 1 in patent application US/20120015991) were synthesized by PharmAkea Inc (San Diego, CA, USA). Sodium pyruvate, NEAA mixture and Penicillin/Streptomycin were purchased from Lonza. L-glutamine was from CellGenix (Portsmouth, NH, USA). Dulbecco’s Modified Eagle’s Medium and fetal bovine serum (FBS) were from Thermo Fisher Scientific (Waltham, MA, USA). Charcoal-stripped FBS was prepared as previously described. In brief, 10 mL of FBS was incubated with 1 g of activated charcoal (Sigma Aldrich) overnight at 4 °C^42^. After centrifugation at 2000 × g for ten minutes, the supernatant was filtered for the experiments. DMSO was from Sigma Aldrich. Mouse primary renal fibroblasts were obtained from Cell Biologics (Chicago, IL, USA). Human umbilical vein endothelial cells (HUVECs) were purchased from Lonza. Recombinant ATX was purchased from R&D systems (Minneapolis, MN, USA).

### Mice

C57BL/6J mice were obtained from Charles River Japan (Atsugi, Japan). Experiments to identify fibroblasts used mice in which all fibroblasts can be identified by their transgenic expression of green fluorescent protein (GFP) driven by the collagen type I, α_2_ promoter (COLI-GFP mice)^27^. These COLI-GFP mice were kindly provided by Dr. Yutaka Inagaki (Tokai University, Isehara, Japan). All experiments used sex- and weight-matched mice at 8–10 weeks of age that were maintained in specific pathogen–free environments. All procedures employed in the animal experiments complied with the standards set out in *the Guidelines for the Care and Use of Laboratory Animals in Takara-machi Campus of Kanazawa University*, and were approved by the Institute for Experimental Animals, Kanazawa University Advanced Research Center (Registration Number: AP-143227).

### Renal fibrosis model

The general procedure of a UUO model is well described elsewhere^43^. Briefly, a flank incision was made and the left ureter ligated with 4-0 silk suture at two points. Obstructed kidneys as well as contralateral ones were harvested from UUO animals for further examinations.

### Inhibitor administration *in vivo*

PAT-048 was dissolved in 0.5% methylcellulose and a dose of 20 mg/kg per mouse was administered by oral gavage to mice once daily. Control mice received equal volumes of 0.5% methylcellulose (vehicle) on the same schedule. PAT-048 or vehicle control was administered from the day of UUO surgery. Kidneys were obtained at the completion of the experiment as described above.

### Tissue preparation

One portion of the renal tissue from each mouse was fixed in 10% buffered formalin (pH 7.2), embedded in paraffin, cut at 4 μm, and used for Mallory-Azan stain.

### RNA analyses

Total cellular RNA was isolated from primary cells using RNeasy Mini Kits (Quiagen, Tokyo, Japan). We isolated total cellular RNA from renal tissue by Trizol reagent (Thermo Fisher Scientific) according to the manufacturer’s protocol. Quantitative real-time PCR analysis using an Applied Biosystems 7900HT Sequence Detection System (Applied Biosystems, Foster City, CA, USA) was performed for the detection of the α_1_ chain of type I procollagen (COLIα_1_), ATX and LPA receptors. Glyceraldehyde-3-phosphate dehydrogenase (GAPDH) and β_2_ microglobulin (β2MG) were used as internal controls. ΔΔCT method was used to calculate relative gene expression of target genes with the internal controls. All the primers we used in this study are listed in Table 1.

**Table 1:** Primer sequences.

Mouse LPA1-F: 5′-ACCTTTGTGGTCATGGTGGT-3′
Mouse LPA1-R: 5′-TCCTGGGTCCAGAACTATGC-3′
Mouse LPA2-F: 5′-TCACTGGTCAATGCAGTGGT-3′
Mouse LPA2-R: 5′-GACACATGCAGCAGAGAAGG-3′
Mouse LPA3-F: 5′-TGGCCAATTTGCTGGTTATT-3′
Mouse LPA3-R: 5′-CGCTTTTTGGTCAAGTTGCT-3′
Mouse LPA4-F: 5′-TGCCTCCCTGTTTGTCTTCT-3′
Mouse LPA4-R: 5′-AAATCAGAGAGGGCCAGGTT-3′
Mouse LPA5-F: 5′-GCTCCAGTGCCCTGACTATC-3′
Mouse LPA5-R: 5′-GTTGAGAGGGAGACCAGTCG-3′
Mouse LPA6-F: 5′-CACTCTGTACGGGTGCATGT-3′
Mouse LPA6-R: 5′-CACTTTGAGGGCACAGATGA-3′
Mouse autotaxin-F: 5′-ATGTACCCTGCCTTCAAACG-3′
Mouse autotaxin-R: 5′-TGACCCCATTCCTTTCTGAC-3′
Mouse aSMA-F: 5′-CTGACAGAGGCACCACTGAA-3′
Mouse aSMA-R: 5′-CATCTCCAGAGTCCAGCACA-3′
Mouse β2MG-F: 5′-CCGAACATACTGAACTGCTACG-3′
Mouse β2MG-R: 5′-CCCGTTCTTCAGCATTTGGA-3′
Human LPA1-F: 5′-GCCATCGTTATGGGTGCTAT-3′
Human LPA1-R: 5′-CTGTAGAGGGGTGCCATGTT-3′
Human LPA2-F: 5′-CCAATCTGCTGGTCATAGCA-3′
Human LPA2-R: 5′-CAGATTGCCGAGCAGGTAGT-3′
Human LPA3-F: 5′-CTCATGGCCTTCCTCATCAT-3′
Human LPA3-R: 5′-TTGTATGCGGAGACAAGACG-3′
Human LPA4-F: 5′-TGCGCTCCCAAGCTATTACT-3′
Human LPA4-R: 5′-GTTCAGAGTTGCAAGGCACA-3′
Human LPA5-F: 5′-TGTTAGCCAACAGCTCCTCA-3′
Human LPA5-R: 5′-CAGCACCAAGCTGTAGACCA-3′
Human LPA6-F: 5′-AGCGTTAACAGCTCCCACTG-3′
Human LPA6-R: 5′-GCACAAACACCATGCTGAAC-3′
Mouse GAPDH: Thermo Fisher Sientific (4352339E)
Human GAPDH: Thermo Fisher Sientific (4326317E)

### Evans blue dye assay of vascular permeability

Vascular permeability was assessed by extravasation of Evans blue dye injected via tail vein using a technique modified from a previously published one^9^. Briefly, Evans blue dye (EBD; 20 mg/kg in 0.9% saline; Sigma Aldrich) was injected intravenously into mice 30 min before they were sacrificed. At the time of sacrifice, blood was collected into a heparinized syringe. Remaining dye in the vessels was washed out by perfusing the mice with 0.9% saline via the left ventricle, and then kidneys were removed and homogenized. Evans blue dye was extracted by the addition of formamide followed by incubation overnight at 60 °C and centrifugation at 5,000 g for 30 min. The absorption of Evans blue in kidney supernatants and plasma was measured at 620 nm using a 96-well plate reader. An Evans blue index was calculated as the ratio of the amount of dye in the kidneys to the plasma dye concentration.

### Localization of renal vascular leak using FITC-conjugated dextran

Fluorescein isothiocyanate (FITC)-conjugated dextran (FITC-dextran; 500000, Sigma Aldrich) was dissolved in normal saline at 7.5 mg/ml, and 150 μl was injected into the tail vein. Kidneys were removed 30 min after the injection and fixed in 10% formalin overnight. Sections of paraffin-embedded tissue were examined to determine the localization of vascular leak using FITC immunostaining as described below. The mean FITC-positive area was determined from the whole area of cortex and outer medulla in the individual complete sagittal kidney section using Image J software (National Institute of Health).

### Immunohistochemical and Immunocytochemical analyses

For the present analysis, formalin-fixed, paraffin-embedded sections were prepared as described above. To identify proliferating fibroblasts, renal sections from COLI-GFP mice were co-stained with anti-GFP monoclonal antibody (Cell Signaling, Danvers, MA, USA) and mouse anti-proliferating cell nuclear antigen (PCNA) monoclonal antibody (Santa Cruz, Dallas, TX, USA), using an M.O.M. kit (Vector laboratories, Burlingame, CA, USA). M.O.M. kit was used to detect mouse primary antibody targeting PCNA by blocking endogenous immunoglobulin in mouse tissue. Antibody-stained cells were visualized using Fluorescein avidin (Vector laboratories) for GFP and Texas-red avidin (Vector laboratories) for PCNA. The number of GFP^+^ and/or PCNA^+^ cells were then counted in ten different fields of specimen and expressed as the mean number ± standard error of the mean (SEM) per high power field (HPF). Rabbit anti-FITC polyclonal antibodies (Agilent, Santa Clara, CA, USA) were used to detect FITC where vascular leak occurred. FITC^+^ areas were visualized by incubating antibody-stained sections with DAB (Nichirei Biosciences, Tokyo, Japan). To determine the co-localization of FITC with ATX, sections were co-stained with mouse anti-FITC monoclonal antibody (Abcam, Cambridge, UK) and rabbit anti-ATX polyclonal antibodies (Thermo Fisher Scientific), using an M.O.M. kit. Antibody-stained cells were visualized using Fluorescein avidin (Vector laboratories) for FITC and Texas-red avidin (Vector laboratories) for ATX. Picro-Sirius red staining of the kidney sections was done according to the manufacturer’s instructions (Polysciences, Warrington, PA, USA). The mean Picro-Sirius red-positive area was determined from the area of cortex and outer medulla in the kidney section using Image J software. Actin polymerization was visualized by immunocytochemical staining for phalloidin in HUVECs. Briefly, HUVECs were stimulated for 30 min with 10 nM ATX added to 10 μM LPC or 10 μM LPC alone. HUVECs were pre-treated with 5 nM PAT-048 or 10 nM AM152 for 30 min. Then, HUVECs were fixed in 4% paraformaldehyde in PBS, followed by the permeabilization with 0.2% Triton X-100 in PBS. Following permeabilization, HUVECs were incubated with rhodamine-phalloidin (Cytoskeleton, Denver, CO) for 30 min. Slides were mounted using Vectashield Mounting Medium with DAPI (Vector laboratories). All the antibodies we used in this study are listed in Table 2.

**Table 2:** List of antibodies for immunohistochemistry and immunoblotting assay.

(Immunohistochemistry)
Rabbit anti-green fluorescent protein monoclonal antibody (D5.1; Cell Signaling)
Mouse anti-proliferating cell nuclear antigen monoclonal antibody (PC10; Santa Cruz)
Rabbit anti-fluorescein isothiocyanate (FITC)/HRP polyclonal antibody (Agilent)
Mouse anti-FITC monoclonal antibody (2A3; Abcam)
Rabbit anti-autotaxin polyclonal antibody (Thermo Fisher Scientific)
(Immunoblotting assay)
Rabbit anti-autotaxin polyclonal antibodies (Thermo Fisher Scientific)
Rabbit anti-GAPDH polyclonal antibody (FL-335; Santa Cruz)

### Western blot analyses

Total cellular lysate was extracted from renal tissue by RIPA buffer (Thermo Fisher Scientific). Cellular lysates were separated by sodium dodecyl sulfate polyacrylamide gel electrophoresis and transferred to polyvinylidene difluoride membranes (Thermo Fisher Scientific). After incubation in blocking buffer containing 5% skim milk (Biorad, Hercules, CA), membranes were incubated with rabbit anti-ATX polyclonal antibodies (Thermo Fisher Scientific) or rabbit anti-GAPDH polyclonal antibody (Santa Cruz) overnight at 4 °C followed by incubation with appropriate biotinylated secondary antibodies. Then, membrane-derived protein bands were detected with an enhanced chemiluminescent substrate (Thermoscientific). Quantification was performed with ImageJ software. All the antibodies we used in this study are listed in Table 2.

### ATX activity assay

Serum were obtained from mice before and ten days after ureteral ligation, especially twenty-four hours after the final administration of PAT-048 or vehicle. Urine samples from ligated kidney or bladder were also obtained ten days after ureteral ligation. ATX activities in these samples were measured as lysophospholipase D activity by measuring choline release from LPC as it is converted to LPA as previously described^21,22^. Briefly, samples were incubated with f.c. 600 μM LPC at 37 °C for 4 hours. Then, the liberated choline was detected by an enzymatic photometric method using choline oxidase, TOOS reagent and horseradish peroxidase (Wako Pure Chemical Industries). ATX activity was expressed as the concentration of liberated choline per minute.

### Fibroblast migration assay

Migration of renal fibroblasts was determined after 4 hours of incubation with 10 μM LPC with or without recombinant ATX using the ChemoTX system (Neuro probe, Gaithersburg, MD, USA) according to the manufacturer’s protocol. Briefly, after media containing stimuli was added to each well of the 96-well plate, a fibronectin-coated membrane with 8 μm pore size was placed on top of the plate such that the media made contact with the bottom side of the membrane. Then, renal fibroblasts were put on the top-side of membrane. Following a 4-hour incubation, the top-side of membrane was scraped with a cotton swab, and the number of renal fibroblasts that migrated to the bottom-side of membrane were counted. A migration index was determined by the ratio of the number of migrated cells in the presence of stimuli to that in control media containing LPC alone. Renal fibroblasts were pre-treated with PAT-048 or AM152 for 30 min prior to the addition of the ATX and LPC.

### Fibroblast proliferation assay

Prior to treatment of renal fibroblasts with reagents, renal fibroblasts were cultured in charcoal-treated serum (0.5%)-containing medium. Renal fibroblast proliferation 24 hours after incubation with 10 μM LPC with or without ATX was determined by a BrdU assay (Roche, Mannheim, Germany), performed according to the manufacture’s protocol. Renal fibroblasts were pre-treated with PAT-048 or AM152 for 30 min prior to the addition of the ATX and LPC.

### Vascular permeability assay

A vascular permeability assay was performed using Cultrex system (Trevigen, Gaithersburg, MD, USA), according to the manufacture’s protocol. Briefly, HUVECs were seeded on a type I collagen-coated membrane in the upper chamber to form a confluent cell monolayer. HUVECs were pre-treated with PAT-048 or AM152 for 30 min followed by the incubation of the ATX and LPC for 30 min. Basal medium was added to the bottom chamber, and then FITC-dextran was added to treated cells in the upper chamber. The amount of FITC-Dextran in the media of bottom chamber was read at 485 nm excitation, 520 nm emission.

### Statistical analyses

Data are expressed as means ± SEMs. Unpaired *t* tests were used for comparisons between two groups, and analysis of variance with *post hoc* Fisher’s test was used for comparisons between more than two groups. *P* values < 0.05 were considered statistically significant.

## Data Availability

All data generated or analysed during this study are included in this published article.
